# Microbiological Characteristics and Resistance Patterns in a Neonatal Intensive Care Unit: A Retrospective Surveillance Study

**DOI:** 10.7759/cureus.56027

**Published:** 2024-03-12

**Authors:** Sameh Kasem, Ahmed Elhadidi, Nuralhuda Omar, Tasnim Dawoud, Omar Abu Sa'da, Aiman Rahmani, Nusrat Khan

**Affiliations:** 1 Pediatrics and Neonatology‎, Tawam Hospital, Al Ain, ARE; 2 Pediatrics and Neonatology, Tawam Hospital, Al Ain, ARE; 3 Pharmacy, Tawam Hospital, Al Ain, ARE

**Keywords:** neonatal sepsis, neonatal intensive care unit, sensitivity, resistance, microbiological

## Abstract

Background and objective: This study aims to assess the prevalence and antimicrobial susceptibility patterns of bacterial infections associated with both early-onset sepsis (EOS) and late-onset sepsis (LOS).

Methodology: This descriptive retrospective surveillance research was conducted on all neonates admitted to the neonatal ICU with bacterial sepsis, where positive cultures were isolated from sterile sites (either cerebrospinal fluid or blood) at Tawam Hospital, Al Ain, Emirate of Abu Dhabi, UAE, from January 2012 and December 2021. Antimicrobial susceptibility analysis was performed.

Results: The incidence of LOS (94.43%) was higher compared to EOS (5.56%). The most prevalent isolates (59.2%) were gram-positive bacteria, with gram-negative bacteria accounting for 40.8%. The leading isolates included coagulase-negative Staphylococci (CONS, 40.98%), Klebsiella (16.04%), Staphylococcus aureus (8.46%), Escherichia coli (8.24%), Pseudomonas (7.57%), and Group B Streptococcus (GBS, 5.12%). CONS were predominant in LOS cases (42.9%), while GBS was the main pathogen in EOS cases (44%).

Conclusions: We observed reduced resistance levels of CONS against ampicillin, benzylpenicillin, clindamycin, erythromycin, fusidic acid, gentamicin, oxacillin, rifampicin, and trimethoprim/sulfa. S. aureus exhibited increased resistance to erythromycin, fusidic acid, gentamicin, and levofloxacin, while E. coli demonstrated decreased resistance against cephalothin, gentamicin, and trimethoprim/sulfa. The antibiotics currently employed empirically appear to provide adequate coverage against the most prevalent bacteria causing early- and late-onset neonatal infections.

## Introduction

Globally, neonatal sepsis is a significant contributor to both morbidity and mortality in neonatal intensive care units (NICUs) [1]. Neonatal sepsis comprises a cluster of clinical presentations that arise from inflammatory reactions triggered by systemic infections, including but not limited to meningitis, septicemia, pneumonia, and bone-related diseases [2]. About 30%-50% of neonatal deaths in developing nations are caused by sepsis. Despite recent advancements in healthcare, the increasing number of neonatal deaths continues to be mostly attributed to delayed diagnosis and inappropriate treatment. Nevertheless, these issues can be prevented with the use of enhanced adjuvant care and proper antimicrobial selection [3]. Despite blood culture being widely regarded as the definitive method for neonatal sepsis, the relatively low frequencies of positive results pose a significant management obstacle [4].

Neonatal sepsis is categorized based on the time at which it manifests: early-onset sepsis (EOS) occurs before 72 hours of age, and late-onset sepsis (LOS) occurs after 72 hours of age. This classification has effects on the suspected organism, potential risk factors, and proposed empirical therapy [5]. Neonatal sepsis is predominantly induced by various species of gram-positive and gram-negative bacteria, with sporadic instances including fungi such as Candida species [6].

As a first-line therapy for the management of suspected neonatal infections, the World Health Organization (WHO) guidelines advise empirical treatment with a combination of ampicillin and gentamicin. In cases where drug-susceptibility testing of bacterial isolates indicates resistance to first-line therapy or when nonresponders develop resistance to first-line therapy, a third-generation cephalosporin is recommended as a second-line therapy. In addition, empirical treatment of EOS with a combination of ampicillin and gentamicin is advised as the initial line of therapy. Diverse empirical therapy suggestions have been put forth for LOS, involving vancomycin paired with gentamicin for nosocomial LOS, ampicillin combined with gentamicin for LOS, and piperacillin-tazobactam for both EOS and LOS [7].

Conversely, the expansion of multi-drug-resistant organisms (MDROs) impedes the development of effective treatments and diminishes the range of adequate antibiotics; thus, institutional guidelines based on the prevalence of local microbes and their sensitivity patterns to antibiotics are required [8]. The scope and resistance of virulent microorganisms have changed year after year due to the broad application of antimicrobial medications [9]. A global threat has emerged in the form of antibiotic resistance. In developing nations, there are increased reports, especially in ICUs, about multi-drug-resistant (MDR) bacteria causing neonatal sepsis. Antibiotic misuse and unnecessary usage, especially of broad-spectrum antibiotics, have been well-established as major contributors to the expansion of drug-resistant strains [10].

Therefore, this study aimed to evaluate the prevalence and antimicrobial-susceptibility patterns of bacterial infections associated with EOS and LOS in our NICU.

## Materials and methods

This retrospective surveillance study was conducted in the NICU at Tawam Hospital. The ethical committee of the institutional review board approved the study. Data were acquired from the microbiological database, and detailed records of all newborns admitted to our unit from January 2012 and December 2021 who developed bacterial sepsis with a positive culture isolated from a sterile site (either blood or cerebrospinal fluid [CSF]), with antimicrobial susceptibility analysis performed. Neonates with an isolated positive culture from a nonsterile site were excluded from this study. For patients with persistent positive cultures, we considered only the first positive culture in our research. Neonatal sepsis was classified into two groups, namely EOS, which occurs 72 hours after birth, and LOS, which takes place after 72 hours of birth [11].

Diagnostic and microbiological criteria

Upon admission to the NICU, newborns underwent peripheral blood cultures if there were indications of suspected sepsis based on their clinical condition or medical history. When a blood culture using a yellow top (pediatric aerobic) tube produced a positive result, the cultures were repeated until achieving a negative result. CSF cultures were obtained if the patient's condition indicated the potential presence of meningitis.

A bacteremia/meningitis episode was defined by the presence of a single positive blood culture and/or CSF culture, according to criteria modified by the Centers for Disease Control and Prevention. However, this requirement was waived in the case of known commensal bacteria, in which case at least two positive cultures were needed, spaced no more than one calendar day apart [12]. Positive cultures of the same bacteria that were repeated within a time frame of less than 14 days were considered to be a single episode. A positive CSF culture was regarded as indicative of an episode, irrespective of a negative blood culture, except for the bacteria mentioned beforehand and in cases involving ventriculostomy or open neural tube malformations.

Our unit's empirical antibiotic treatment regimens were Penicillin G and gentamicin for early-onset bacteremia or flucloxacillin and gentamicin for late-onset bacteremia. Vancomycin was administered in cases where bacterial sensitivity recommended its use when clusters of gram-positive cocci were seen before the definitive diagnosis of the organism or where there was an elevated likelihood of central line infection. In cases when there is a high level of suspicion or confirmation of meningitis, a third-generation cephalosporin was provided to ensure adequate coverage.

Regarding the antibiotic sensitivity test, the antibiotics used for gram-negative bacilli included amikacin, augmentin, aztreonam, cefepime, cefotaxime/ceftazidime, cefuroxime, cephalothin, ciprofloxacin, gentamicin, imipenem/meropenem, levofloxacin, piperacillin/tazobactam, tobramycin, and trimethoprim/sulfamethoxazole. The gram-positive bacteria antibiotics used included ampicillin, benzylpenicillin, clindamycin, erythromycin, fusidic acid, gentamicin, levofloxacin, linezolid, oxacillin, rifampicin, tobramycin, trimethoprim/sulfamethoxazole, and vancomycin. All discs were obtained from Oxoid, England. Interpretation of the inhibition zones associated with different antibiotics was conducted in accordance with Clinical and Laboratory Standards Institute (CLSI) recommendations [2]. MDR bacteria can be identified as bacteria that exhibit resistance to a minimum of one agent from three or more of the evaluated antimicrobial classes [13].

Statistical analysis

Statistical analysis was done using IBM SPSS Statistics for Windows, Version 28 (IBM Corp., Armonk, NY). Qualitative variables were presented as frequency and percentage (%) and analyzed using the chi-square test or Fisher's exact test when appropriate. A two-tailed *P*-value < 0.05 was considered statistically significant.

## Results

A total of 449 septic episodes with positive blood or CSF cultures fulfilled our inclusion criteria during the specified period, among which 25 (5.56%) were EOS episodes and 424 (94.43%) were LOS episodes. Table 1 shows the incidence of EOS and LOS episodes throughout the 10-year period, where we found that the incidence of LOS was higher compared to EOS.

**Table 1:** Incidence of EOS and LOS over a 10-year period (2012-2021). Data were presented as frequency (%). EOS, early-onset sepsis; LOS, late-onset sepsis

Year	Number of live births	Number of admissions	Number of EOS	Incidence/1,000 live births	Incidence/1,000 admissions	Number of LOS	Incidence/1,000 live births	Incidence/1,000 admissions
2012	4,107	519	-	-	-	42	1.02%	8.09%
2013	3,837	579	5	0.13%	0.86%	24	0.62%	4.14%
2014	3,756	588	1	0.02%	0.17%	77	2.05%	13.09%
2015	3,756	568	1	0.02%	0.17%	41	1.09%	7.21%
2016	3,917	582	2	0.051%	0.34%	53	1.40%	9.45%
2017	3,853	598	4	0.10%	0.66%	42	1.09%	7.02%
2018	3,194	582	4	0.12%	0.68%	34	1.06%	5.84%
2019	3,068	562	3	0.09%	0.53%	38	1.23%	6.76%
2020	3,286	685	1	0.03%	0.14%	30	0.91%	4.37%
2021	3,007	671	4	0.13%	0.59%	43	1.42%	6.40%

A total of 449 neonates with positive culture results were documented in the laboratory records during the study period. The most common isolates comprised 266 (59.2%) gram-positive bacteria, while 183 (40.8%) were gram-negative bacteria. The predominant isolates were coagulase-negative staphylococci (CONS) with 184 cases (40.98%), followed by Klebsiella with 72 cases (16.04%), Staphylococcus aureus with 38 cases (8.46%), Escherichia coli with 37 cases (8.24%), Pseudomonas with 34 cases (7.57%), and group B Streptococcus (GBS) with 23 cases (5.12%). GBS emerged as the primary pathogen in EOS with 11 cases (44%), while CONS was the predominant pathogen in LOS cases, accounting for 182 (42.9%; Table 2).

**Table 2:** Frequency of causative microorganisms in EOS and LOS over a 10-year period (2012-2021). Data were presented as frequency (%). GBS, Group B streptococcus; CONS, coagulase-negative staphylococci; EOS, early-onset sepsis; LOS, late-onset sepsis

Microorganism	EOS	LOS	*n* (%)
	25 (5.56%)	424 (94.43%)	449 (100%)
GBS	11 (44%)	12 (2.83%)	23 (5.12%)
Staphylococcus aureus	2 (8%)	36 (8.49%)	38 (8.46%)
CONS	2 (8%)	182 (42.9%)	184 (40.98%)
Enterococci	0 (0%)	17 (4%)	17 (3.79%)
Other Streptococci	1 (4%)	3 (0.70%)	4 (0.89%)
Pseudomonas	0 (0%)	34 (8.01%)	34 (7.57%)
Escherichia coli	6 (24%)	31 (7.1%)	37 (8.24%)
Klebsiella	1 (4%)	71 (16.74%)	72 (16.04%)
Acinetobacter	0 (0%)	5 (1.17%)	5 (1.11%)
Enterobacter	0 (0%)	18 (4.24%)	18 (4.01%)
Stenotrophomonas maltophilia	0 (0%)	10 (2.35%)	10 (2.23%)
Citrobacter	0 (0%)	1 (0.24%)	1 (0.22%)
Haemophilus influenzae	2 (8%)	0 (0%)	2 (0.45%)
Morganella morganii	0 (0%)	1 (0.24%)	1 (0.22%)
Stenotrophomonas	0 (0%)	3 (0.70%)	3 (0.67%)

The antibiotic resistance patterns of the predominant gram-positive bacteria from 2012 to 2021 are outlined in Table 3. CONS exhibited high resistance levels to ampicillin, penicillin, oxacillin, gentamicin, erythromycin, and fusidic acid, and relatively lower resistance to vancomycin, rifampicin, and co-trimoxazole. S. aureus demonstrated the highest resistance to penicillin and ampicillin, followed by erythromycin, with no observed resistance to vancomycin and lower resistance to rifampicin and clindamycin. Enterococci displayed the highest resistance to erythromycin, followed by gentamicin, with no resistance to ampicillin, and lower resistance to vancomycin and penicillin. GBS showed the highest resistance to erythromycin, lower resistance to penicillin, and no resistance to vancomycin and ampicillin. Other streptococci did not exhibit resistance to any of the antibiotics used.

**Table 3:** Resistance of gram-positive cocci organisms to antibiotics. Data were presented as frequency (%). GBS, Group B Streptococcus; CONS, coagulase-negative staphylococci

	CONS (*n *= 184)	Staphylococcus aureus (*n *= 38)	Enterococci *(n *= 17)	GBS (*n *= 23)	Other streptococci (*n *= 4)
Oxacillin	171 (92.93%)	14 (36.84%)	-	-	-
Gentamicin	163 (88.59%)	10 (26.32%)	6 (35.29%)	-	-
Vancomycin	1 (0.54%)	0 (0%)	1 (5.88%)	0 (0%)	0 (0%)
Fusidic acid	158 (85.87%)	11 (28.95%)	-	-	-
Co-trimoxazole	24 (13.04%)	8 (21.05%)	-	-	-
Rifampicin	9 (4.89%)	2 (5.26%)	-	-	-
Clindamycin	57 (30.98%)	4 (10.53%)	-	-	0 (0%)
Penicillin	180 (97.83%)	33 (86.84%)	1 (5.88%)	1 (4.35%)	0 (0%)
Erythromycin	158 (85.87%)	16 (42.11%)	16 (94.12%)	16 (69.57%)	0 (0%)
Ampicillin	181 (98.37%)	32 (84.21%)	0 (0%)	0 (0%)	0 (0%)

The most prevalent gram-positive isolates during the study period, spanning from 2012 to 2021, were CONS and S. aureus. We observed a significant decrease in resistance levels of CONS against ampicillin, benzylpenicillin, clindamycin, erythromycin, fusidic acid, gentamicin, oxacillin, rifampicin, and trimethoprim/sulfamethoxazole (*P *< 0.05). Furthermore, CONS did not develop resistance to levofloxacin, linezolid, tobramycin, and vancomycin during the same period.

In contrast, S. aureus exhibited a significant increase in resistance levels against erythromycin, fusidic acid, gentamicin, and levofloxacin (*P *< 0.05). Additionally, there were insignificant increases in resistance against ampicillin, benzylpenicillin, clindamycin, and oxacillin, with constant resistance against trimethoprim/sulfamethoxazole observed from 2012 to 2021. Notably, S. aureus did not develop resistance to linezolid, rifampicin, tobramycin, and vancomycin during the same period (Table 4).

**Table 4:** Trends in antibiotic resistance of the most prevalent gram-positive cocci isolates from neonatal sepsis patients (2012-2021). Data were presented as frequency (%). ^*^Statistically significant at *P*-value < 0.05. GBS, Group B streptococcus; CONS, coagulase-negative staphylococci

	CONS (*n *= 184)		*P*-value	Staphylococcus aureus (*n *= 38)		*P*-value
	2012-2017	2018-2021		2012-2017	2018-2021	
Ampicillin	102 (55.4%)	15 (8.2%)	<0.001*	16 (42.1%)	13 (34.2%)	0.637
Benzylpenicillin	107 (58.2%)	15 (8.2%)	<0.001*	19 (50%)	14 (36.8%)	0.355
Clindamycin	36 (19.6%)	7 (3.8%)	<0.001*	3 (7.9%)	1 (2.6%)	0.615
Erythromycin	87 (47.3%)	13 (7.1%)	<0.001*	3 (7.9%)	13 (34.2%)	0.009*
Fusidic acid	76 (41.3%)	13 (7.1%)	<0.001*	0 (0%)	11 (28.9%)	<0.001*
Gentamicin	89 (48.4%)	12 (6.5%)	<0.001*	0 (0%)	9 (23.7%)	0.002*
Levofloxacin	4 (2.2%)	0 (0%)	0.123	1 (2.6%)	8 (21.1%)	0.028*
Linezolid	0 (0%)	0 (0%)	-	0 (0%)	0 (0%)	-
Oxacillin	102 (55.4%)	13 (7.1%)	<0.001*	9 (23.7%)	5 (13.2%)	0.375
Rifampicin	8 (4.3%)	1 (0.5%)	0.037*	0 (0%)	0 (0%)	-
Tobramycin	2 (1.1%)	0 (0%)	0.499	0 (0%)	0 (0%)	-
Trimethoprim/Sulfa	13 (7.1%)	3 (1.6%)	0.019*	4 (10.5%)	4 (10.5%)	1.0
Vancomycin	1 (0.5%)	0 (0%)	1.0	0 (0%)	0 (0%)	-

Table 5 illustrates the antibiotic resistance patterns of the predominant gram-negative bacteria from 2012 to 2021. E. coli displayed a high level of resistance against co-trimoxazole, followed by cefuroxime, cefotaxime/ceftazidime, cefepime, and gentamicin, with a lower level of resistance against piperacillin/tazobactam and no resistance against meropenem. Klebsiella exhibited the highest resistance to cefuroxime, followed by cefotaxime/ceftazidime, with lower resistance observed against gentamicin and piperacillin/tazobactam, and no resistance against meropenem. Pseudomonas demonstrated a low level of resistance to the antibiotics used, with no resistance against gentamicin and meropenem. Acinetobacter showed the highest level of resistance against cefuroxime only, with no resistance against other antibiotics.

**Table 5:** Resistance of gram-negative rods to antibiotics. Data were presented as frequency (%).

	Escherichia coli (*n *= 37)	Klebsiella (*n *= 72)	Pseudomonas (*n *= 34)	Acinetobacter (*n *= 5)	Haemophilus influenzae (*n *= 2)	Morganella morganii (*n *= 1)	Serratia (*n *= 10)	Enterobacter (*n *= 18)	Citrobacter (*n *= 1)
Gentamicin	12 (32.4%)	1 (1.4%)	0 (0%)	0 (0%)	-	0 (0%)	0 (0%)	0 (0%)	0 (0%)
Cefotaxime/Ceftazidime	13 (35.1%)	15 (20.8%)	2 (5.9%)	0 (0%)	0 (0%)	1 (100%)	10 (100%)	15 (83.3%)	0 (0%)
Cefepime	13 (35.1%)	8 (11.1%)	2 (5.9%)	0 (0%)	0 (0%)	1 (100%)	10 (100%)	15 (83.3%)	0 (0%)
Meropenem	0 (0%)	0 (0%)	0 (0%)	0 (0%)	0 (0%)	0 (0%)	0 (0%)	0 (0%)	0 (0%)
Aztreonam	-	-	-	-	-	-	-	-	0
Ciprofloxacin	-	-	-	0 (0%)	0 (0%)	-	-	-	-
Levofloxacin	-	-	-	0 (0%)	0 (0%)	-	-	-	-
Piperacillin/tazobactam	3 (8.11%)	4 (5.6%)	1 (2.9%)	0 (0%)	-	0 (0%)	0 (0%)	4 (22.2%)	0 (0%)
Amoxicillin/clavulanate	9 (24.3%)	8 (11.1%)	-	-	0 (0%)	-	-	18 (100%)	0 (0%)
Co-trimoxazole	22 (59.5%)	12 (16.7%)	-	0 (0%)	1 (50%)	0 (0%)	0 (0%)	2 (11.1%)	0 (0%)
Cefuroxime	15 (40.5%)	21 (29.2%)	-	5 (100%)	0	1 (100%)	10 (100%)	18 (100%)	1 (100%)

Less common gram-negative rods, such as Haemophilus influenzae (which developed resistance against co-trimoxazole only), Morganella morganii, and Serratia, exhibited high resistance against cefotaxime/ceftazidime, cefepime, and cefuroxime. Enterobacter displayed a high level of resistance against amoxicillin/clavulanate, and cefuroxime, followed by cefotaxime/ceftazidime and cefepime. Citrobacter showed resistance against cefuroxime only. In conclusion, meropenem was identified as the most effective antibiotic against the isolated gram-negative rods.

The most prevalent gram-negative isolates during the study period from 2012 to 2021 were Klebsiella, E. coli, and Pseudomonas, respectively. We observed that E. coli exhibited significantly decreased levels of resistance against cephalothin, gentamicin, and trimethoprim/sulfamethoxazole (*P *= 0.010, 0.003, and 0.001, respectively). Additionally, E. coli showed insignificant changes in resistance levels against augmentin, cefepime, cefotaxime/ceftazidime, and cefuroxime and did not develop resistance to amikacin, aztreonam, ciprofloxacin, imipenem/meropenem, levofloxacin, piperacillin/tazobactam, and tobramycin.

Both Klebsiella and Pseudomonas exhibited insignificant changes in resistance levels against all tested antibiotics from 2018 to 2021 compared to 2012 to 2017. However, Klebsiella did not develop resistance to amikacin, aztreonam, ciprofloxacin, gentamicin, imipenem/meropenem, and levofloxacin. Pseudomonas did not develop resistance to amikacin, aztreonam, cefuroxime, cephalothin, ciprofloxacin, gentamicin, imipenem/meropenem, and tobramycin. An insignificant development of resistance was observed against augmentin, cefepime, cefotaxime/ceftazidime, piperacillin/tazobactam, and trimethoprim/sulfamethoxazole (Table 6).

**Table 6:** Trends in antibiotic resistance of the most prevalent gram-negative rod isolates from neonatal sepsis patients (2012-2021). Data were presented as frequency (%). ^*^Statistically significant at *P*-value < 0.05.

	Escherichia coli (*n *= 37)		*P*-value	Klebsiella (*n *= 72)		*P*-value	Pseudomonas (*n *= 34)		*P*-value
	2012-2017	2018-2021		2012-2017	2018-2021		2012-2017	2018-2021	
Amikacin	4 (10.8%)	0 (0%)	0.115	2 (2.8%)	0 (0%)	0.496	0 (0%)	0 (0%)	-
Augmentin	6 (16.2%)	3 (8.1%)	0.479	4 (5.6%)	2 (2.8%)	0.681	0 (0%)	2 (5.9%)	0.492
Aztreonam	0 (0%)	0 (0%)	-	0 (0%)	0 (0%)	-	1 (2.9%)	0 (0%)	1.00
Cefepime	1 (2.7%)	4 (10.8%)	0.357	0 (0%)	4 (5.6%)	0.120	0 (0%)	2 (5.9%)	0.492
Cefotaxime/Ceftazidime	9 (24.3%)	4 (10.8%)	0.221	2 (2.8%)	7 (9.7%)	0.166	0 (0%)	2 (5.9%)	0.492
Cefuroxime	10 (27%)	4 (10.8%)	0.136	7 (9.7%)	11(15.3%)	0.450	0 (0%)	0 (0%)	-
Cephalothin	16 (43.2%)	5 (13.5%)	0.010*	6 (8.3%)	11 (15.3%)	0.302	0 (0%)	0 (0%)	-
Ciprofloxacin	5 (13.5%)	0 (0%)	0.054	0 (0%)	0 (0%)	-	0 (0%)	0 (0%)	-
Gentamicin	11 (29.7%)	1 (2.7%)	0.003*	1 (1.4%)	0 (0%)	1.0	0 (0%)	0 (0%)	-
Imipenem/Meropenem	0 (0%)	0 (0%)	-	0 (0%)	0 (0%)	-	0 (0%)	0 (0%)	-
Levofloxacin	0 (0%)	0 (0%)	-	0 (0%)	0 (0%)	-	0 (0%)	0 (0%)	-
Piperacillin/Tazobactam	3 (8.1%)	0 (0%)	0.240	2 (2.8%)	2 (2.8%)	1.0	0 (0%)	1 (2.9%)	1.00
Tobramycin	0 (0%)	0 (0%)	-	0 (0%)	0 (0%)	-	0 (0%)	0 (0%)	-
Trimethoprim/Sulfa	16 (43.2%)	3 (8.1%)	0.001*	5 (6.9%)	7 (9.7%)	0.763	0 (0%)	3 (8.8%)	0.239

Table 7 depicts the trend of resistant microorganisms over the 10 years from 2012 to 2021. The detection of methicillin-resistant Staphylococcus aureus (MRSA) decreased in the last three years, with four identifications in 2019 and only one in 2021. Pseudomonas with MDROs was not isolated in the previous four years of our study. There was a significant increase in the isolation of Enterobacteriaceae resistant to third-generation cephalosporin or extended-spectrum beta-lactamase (ESBL) in the last year, 2021, accounting for seven cases (15.91%). Enterobacteriaceae resistant to carbapenem were not reported throughout the study period.

**Table 7:** Distribution trends of resistant microorganisms over a 10-year period (2012-2021). Data were presented as frequency (%). MORO, multi-drug-resistant organism; MRSA, methicillin-resistant Staphylococcus aureus; ESBL, extended-spectrum beta-lactamase

	MRSA	Pseudomonas MDRO	Enterobacteriaceae resistant to third-generation cephalosporin or ESBL	Enterobacteriaceae resistant to carbapenem
2012	3 (23.08%)	0 (0%)	3 (6.82%)	0 (0%)
2013	0 (0%)	0 (0%)	0 (0%)	0 (0%)
2014	3 (23.08%)	2 (50%)	8 (18.18%)	0 (0%)
2015	2 (15.38%)	0 (0%)	5 (11.36%)	0 (0%)
2016	0 (0%)	0 (0%)	8 (18.18%)	0 (0%)
2017	0 (0%)	2 (50%)	9 (20.45%)	0 (0%)
2018	0 (0%)	0 (0%)	3 (6.82%)	0 (0%)
2019	4 (30.77%)	0 (0%)	1 (2.27%)	0 (0%)
2020	0 (0%)	0 (0%)	0 (0%)	0 (0%)
2021	1 (7.69%)	0 (0%)	7 (15.91%)	0 (0%)
Total	13	4	44	0

## Discussion

Neonates, being immunocompromised individuals, are susceptible to infections that can result in significant morbidity and mortality. The American Academy of Pediatrics recommends the initial treatment of neonatal sepsis and meningitis with a combination of ampicillin and gentamicin. Our study revealed that 13% of early-onset gram-negative organisms were resistant to gentamicin, compared to 15% resistance to cefotaxime, 7% to amikacin, and 10% to meropenem [14]. Due to the rapid evolution of bacterial resistance to β-lactam antibiotics, meropenem is typically reserved for treating the most severe and resistant infections [15]. The most common organism causing early-onset disease was GBS (29.2%, 0.38/1,000 live births), while CONS were the most prevalent in late-onset disease (51%) [14].

In our study, we observed that LOS incidence was higher than EOS (94.43% vs. 5.56%). Among LOS episodes, the most prevalent isolates were 266 (59.2%) gram-positive bacteria, whereas 183 (40.8%) were gram-negative bacteria. The predominant isolates were CONS (184, 40.98%), followed by Klebsiella (72, 16.04%), S. aureus (38, 8.46%), E. coli (37, 8.24%), Pseudomonas (34, 7.57%), and GBS (23, 5.12%). GBS was the predominant pathogen in EOS (44%), while CONS was the predominant pathogen in LOS cases (42.9%).

Consistent with earlier studies, Mintz et al. [16] showed that CONS was the predominant bacterium responsible for late-onset bacteremia, accounting for 60.5% of cases [17,18]. Two published studies reported GBS as the most prevalent organism in EOS cases (60%), followed by CONS (6%) and Klebsiella (4%). CONS was the most prevalent isolate (34%) among their LOS cases, consistent with Almohammady et al.'s result (31%) [2]; however, the prevalence of Klebsiella varied between Almohammady et al.'s findings (41.5%) and their results (22.8%) [19,20].

Regarding the resistance of gram-positive cocci organisms to antibiotics, we found that CONS showed a high level of resistance against ampicillin, penicillin, oxacillin, gentamicin, erythromycin, and fusidic acid and less resistance against vancomycin, rifampicin, and co-trimoxazole. S. aureus showed the highest resistance to penicillin, followed by ampicillin and erythromycin. E. coli exhibited a high level of resistance against co-trimoxazole, followed by cefuroxime, cefotaxime/ceftazidime, cefepime, and gentamicin, with less resistance against piperacillin/tazobactam. Klebsiella displayed the highest resistance to cefuroxime, followed by cefotaxime/ceftazidime, with less resistance observed against gentamicin and piperacillin/tazobactam. Pseudomonas showed a low level of resistance to the used antibiotics, with no resistance against gentamicin and meropenem. In conclusion, meropenem was identified as the most effective antibiotic against the isolated gram-negative rods.

Sorsa et al. [21] reported the identification of bacteria in 88 (29.4%) of the investigated blood cultures. The most frequently found bacteria were S. aureus (16, 18%), CONS (22, 25%), and E. coli (E. Coli) 18 (20.5%). The respective resistance rates of CONS and S. aureus to ampicillin were 20 (91%) and 11 (69%). The prevalence of resistance among E. coli to gentamycin and ampicillin was 10 (55.6%) and 12 (66.7%), respectively. In contrast, the resistance rates of Klebsiella spp. to these two first-line antibiotics were considerably higher (10, 91%, and 9, 82%, respectively). Similarly, gram-negative and gram-positive bacterial isolates exhibited significant resistance to third-generation cephalosporins, with 63 (72%) of the isolated bacteria demonstrating resistance to multiple drugs. On the other hand, the susceptibility patterns of gram-positive bacteria isolates to third-line antibiotics such as clindamycin, ciprofloxacin, and vancomycin were superior to those of gram-negative isolates to amikacin and ciprofloxacin.

Sharma et al. [22] observed that Klebsiella showed susceptibility to amikacin (20%) and gentamicin (0%), but exhibited lower susceptibility (30%) to levofloxacin. In a study by the Gulf Cooperation Council (GCC), more than half of Klebsiella isolates and 43% of all gram-negative isolates from LOS neonates were resistant to third-generation cephalosporins [20]. Higher susceptibilities of Klebsiella isolates to amikacin (89%), gentamicin (83%), and cephalosporins (83%-85%) were reported by Al-Matary et al. [23], reflecting potentially more controlled antibiotic usage.

Enterobacter, as studied by Almohammady et al. [2], exhibited 100% susceptibility to levofloxacin, 67% susceptibility to amikacin and ciprofloxacin, and 100% resistance to imipenem. Sharma et al. [22] reported 100% susceptibility of Enterobacter to levofloxacin, gentamicin, and imipenem, with 25% susceptibility to amikacin and ciprofloxacin. Al-Matary et al. [23] found Enterobacter to be 100% sensitive to amikacin and 83% sensitive to gentamycin and cefepime. These variations among studies may stem from differences in antibiotic preferences in different communities, influencing distinct patterns of antibiotic resistance development.

Bromiker et al. [14] demonstrated resistance patterns of staphylococci and gram-negative organisms to key antibiotics. CONS showed 73% resistance to methicillin, with no resistance to vancomycin. Most gram-positive organisms, excluding staphylococci, were susceptible to ampicillin, and all were susceptible to vancomycin. For gram-negative isolates, 74% were resistant to ampicillin. E. coli exhibited a low susceptibility (38%) to ampicillin, while 83% of isolates were susceptible to gentamicin. Pseudomonas species showed resistance to gentamicin (36%), mezlocillin, and meropenem, with lower resistance to ceftazidime (18%) and amikacin (9%). Overall, there was a trend of increasing resistance of nosocomially acquired gram-negative organisms to β-lactam and aminoglycoside antibiotics compared to vertically acquired ones, with statistical significance observed only for ampicillin and mezlocillin (*P *< 0.05 and *P *< 0.01, respectively). Fahmey [24] reported that of 673 screened neonates, 138 (20.5%) had positive blood cultures, confirming early-onset neonatal sepsis (EONS). Among recovered isolates, 86.2% were gram-negative pathogens, with Klebsiella pneumoniae (42.8%), Enterobacter cloacae (22.5%), and E. coli (13.8%) being the most common. The most frequent gram-positive microorganism was S. aureus, constituting only 8.7% of isolates. All Klebsiella isolates and 93% of Enterobacter isolates were resistant to ampicillin. Gram-negative pathogens displayed maximum overall sensitivity to imipenem, cefepime, and ciprofloxacin, while gram-positive isolates were most susceptible to vancomycin, imipenem, and piperacillin. Species were generally resistant to ampicillin. Shahian et al. [25] found that approximately 33% of Klebsiella isolates were sensitive to ampicillin, while high susceptibility to imipenem (98%) and ciprofloxacin (96%) was reported by Macharashvili et al. [26]. Due to the rapid evolution of bacterial resistance to β-lactam antibiotics, imipenem is often reserved for treating the most severe and resistant infections [14]. Moreover, prior use of antibacterial drugs, particularly cephalosporins, ampicillin, and gentamicin, along with prolonged exposure, is associated with a high prevalence of MDR bacteria [27]. MDR bacteria are not confined to hospitals; they are also prevalent in the community environment, particularly in the Middle East, due to the excessive use and over-the-counter availability of antibiotics [28].

We observed that CONS exhibited significantly decreased levels of resistance against ampicillin, benzylpenicillin, clindamycin, erythromycin, fusidic acid, gentamicin, oxacillin, rifampicin, and trimethoprim/sulfa. Conversely, S. aureus displayed significantly increased levels of resistance against erythromycin, fusidic acid, gentamicin, and levofloxacin. E. coli demonstrated significantly decreased levels of resistance against cephalothin, gentamicin, and trimethoprim/sulfa. Both Klebsiella and Pseudomonas showed insignificant changes in resistance levels against all tested antibiotics from 2018 to 2021 compared to 2012-2017.

In a study by Tessema et al. [29], E. coli, the predominant gram-negative bacteria, exhibited the highest overall proportion of resistance to ampicillin, followed by ampicillin-sulbactam and piperacillin during the study period. Furthermore, E. coli displayed rising trends of resistance against ampicillin, ampicillin-sulbactam, piperacillin, and cefuroxime during the study period. However, it is noteworthy that E. coli demonstrated significantly declining trends of resistance to ciprofloxacin and levofloxacin over the years. Additionally, all E. coli isolates were susceptible to gentamicin, amikacin, colistin, fosfomycin, imipenem, piperacillin-tazobactam, and tobramycin, consistent with previous research [30].

Regarding the distribution of resistant microorganisms over 10 years, MRSA detection decreased in the last three years, being identified four times in 2019 and only once in 2021. Pseudomonas MDROs were not isolated in the last four years of our study. There has been a significant increase in the isolation of Enterobacteriaceae resistant to third-generation cephalosporin or ESBL in the last year, 2021, with a prevalence of 7 (15.91%). Enterobacteriaceae resistant to carbapenem were not reported during the study period.

The limitations of this study include its retrospective nature, focus on a single center in the United Arab Emirates, and the absence of confirmatory cultures for potential contaminant pathogens. The potential presence of anaerobic bacteria, occasionally leading to neonatal sepsis, was not considered. The isolation of CONS in early neonatal sepsis might be attributed to contamination during blood sample collection. The absence of molecular methods and limited availability of biochemical assays precluded the determination of extended beta-lactamase-resistant strains.

Conducting periodic evaluations of antibiotic empiric protocols is imperative in light of emerging bacterial resistance. To ensure the appropriate use of antibiotics, potent options such as carbapenems should be reserved for critical situations. Antibiotic stewardship is crucial to ensure the prudent application of antimicrobial therapy. Nevertheless, prevention remains the most crucial strategy for managing infections in EOS and LOS.

## Conclusions

Our study underscores the critical importance of a holistic and nuanced approach to antimicrobial resistance (AMR) within NICUs. Recognizing the complex nature of AMR, we acknowledge that while reducing the usage of certain antibiotics can contribute to a decrease in resistance levels over time, the elimination of drug resistance is a multifaceted challenge that extends beyond simply ceasing the use of specific antimicrobials. The persistence of resistance mechanisms and the potential for horizontal gene transfer among bacteria necessitate comprehensive strategies aimed at AMR management.

To this end, our findings advocate for the implementation of robust antimicrobial stewardship programs (ASPs) tailored to NICU settings, emphasizing the judicious selection and use of antibiotics. Infection prevention and control measures are paramount in curtailing the spread of resistant organisms, complemented by ongoing surveillance of antimicrobial use and resistance patterns. Educating healthcare professionals, patients, and the wider community about the prudent use of antibiotics is crucial in fostering responsible antibiotic practices. Collectively, these strategies represent key components in our arsenal against AMR, aiming to preserve the efficacy of existing antibiotics, enhance neonatal patient care, and mitigate the emergence and spread of resistance within and beyond NICU environments.
